# The Ancova model for comparing two groups: a tutorial emphasizing statistical distribution theory

**DOI:** 10.3389/fpsyg.2025.1600764

**Published:** 2025-05-15

**Authors:** Wolf Schwarz

**Affiliations:** Department of Psychology, University of Potsdam, Potsdam, Germany

**Keywords:** analysis of covariance, conditional vs. marginal means, gain scores, noncentral *F*-distribution, randomized design, observational study

## Abstract

The analysis of covariance (Ancova) is a widely used statistical technique for the comparison of groups with respect to a quantitative dependent variable in such a way that the comparison takes into account concomitant differences in a quantitative covariate. Despite its widespread use, some of the main features of this technique have remained elusive, contentious, or misconceived in applied settings. For example, some authors claim that the validity of an Ancova depends on the assumption that the expected value of the covariate is the same for all participants, or that the adjusted mean difference evaluated in an Ancova has a useful interpretation as the difference between the mean change scores of each group, whereas these claims are disputed by other authors. I suggest that these issues are best addressed and settled in the context of the underlying exact sampling distribution theory since significance statements, effect size estimates, and statistical power all derive directly from the statistical sampling distribution theory implied by the Ancova model. The distributional approach also clarifies the central distinction between conditional and marginal means, and the way in which various study designs (controlled, randomized, observational) affect and modify conclusions derived from an Ancova. The tutorial provides an explicit distributional account of the standard Ancova model to compare two independent groups; it clarifies the assumptions underlying the Ancova model, the nature and limitations of the conclusions it provides, and corrects some common misconceptions associated with its applications.

## 1 Introduction

Empirical studies often aim to compare two or more treatments applied to separate groups. A standard technique for this purpose is the analysis of variance (Anova), which in the case of two independent groups is equivalent to an independent samples *t*−test. In these techniques the mean variability within each group defines and delimits the resolution with which differences between the groups can be identified. It is therefore natural to consider ways to refine this resolution so that the comparison becomes more sensitive, and fewer units are required to detect a given difference. Effective techniques to achieve this aim are, for example, blocking, or the use of repeated measures from the same unit (for general background, see, e.g., Maxwell et al., 2018, ch. 9; Kutner et al., 2004, ch.s 26–29).

In the case of two groups, blocking typically uses a *covariate* to form pairs (blocks) of units that are as similar as possible with respect to the covariate. Treatments are then randomly assigned to one or the other member of any given pair; this procedure serves to make the two groups more similar on average. Blocking leads to more sensitive comparisons of the treatments, as potentially relevant differences in the covariate between the units are balanced more systematically than under unconstrained randomization. However, the technique of blocking requires that the covariate is available before the units are assigned to treatment groups, and with quantitative covariates it ignores the residual variability of the covariate within blocks (for a discussion of further limitations of blocking, see Maxwell et al., 2018, ch. 9; Schwarz and Reike, 2018; Schwarz, 2008). A widely used technique that makes use of all the quantitative information in the covariate is the analysis of covariance (Ancova; Huitema, 2011; Kutner et al., 2004, ch. 22; Schneider et al., 2015; Senn, 2006; Vickers and Altman, 2001). I illustrate typical Ancova applications by two examples, Study A and B.

### 1.1 Study A

To compare how much time it takes consumers to empty a straight (cylindrical) vs. curved (truncated cone) glass of beer (volume in both cases 12 fl oz), naive subjects were randomly assigned to two treatment groups defined by glass shape (for a detailed study, see Attwood et al., 2012). As a potentially relevant covariate, a questionnaire score was obtained at the end of the study from all participants which assessed the urge to consume alcoholic beverages so as to control the comparison related to glass shape for potential base differences of drinking habits. Here, the treatment variable defining the groups is the shape of the glass, the dependent variable is the drinking time, and the covariate is the questionnaire score.

### 1.2 Study B

The high jump performance of randomly selected male and female high-school graduates were compared. As a covariate, the body height of each graduate was recorded, so as to control the comparison related to gender for potential differences on this variable. Here, the variable defining the groups is gender, the dependent variable is the performance (maximum height jumped over) of the graduate, and the covariate is his/her body height.

Note that in neither Study A nor B did the researcher exert any *direct* control over the covariate. Rather, the covariate values arise with the specific participants who are randomly sampled, and these values would clearly change in any replication of either study. Study A uses a randomized design; consequently, in many hypothetical replications of that study the long-run average of the covariate would not be expected to differ between the two groups, although of course in each individual sample, the means would typically be different. In contrast, even though the male and female graduates were randomly selected in Study B, the assignment of a particular participant to the two gender groups is not under the control of the researcher. Therefore, any covariate that differs between these two groups could be at least partially responsible for the differences in the dependent variable. When using body height as a covariate, the investigator asks whether there would be a systematic difference in the high jump performance of male vs. female graduates if these graduates were the same height. The question is clearly counterfactual, because it is well-established that male and female graduates differ systematically in height. Addressing this counterfactual aspect clearly requires some form of model-related assumptions in order to arrive at valid interpretations of an Ancova in observational designs (e.g., Huitema, 2011, ch. 8; Lord, 1967).

Expositions of the Ancova typically represent its central assumptions indirectly, for example in graphical form with Venn or flow diagrams, by focusing on detailed numerical calculations within a single specific sample, or by analogy to regression modeling. It is plausible that this indirect way of presenting the Ancova model has contributed to various ambiguities and controversies regarding, for example, the assertion that for an Ancova to be valid all participants must have the same expected value of the covariate (e.g., Schneider et al., 2015), or that (if the dependent variable and the covariate are commensurate) the adjusted mean difference evaluated in an Ancova represents the difference between the mean change scores of each group (e.g., Vickers and Altman, 2001). Surprisingly few accounts in the applied Ancova literature are based on the actual sampling distributions associated with the Ancova (for some exceptions at a technically advanced level, see Schneider et al., 2015; Shieh, 2017, 2021). This is unfortunate, because the central topics associated with an Ancova are best understood directly in terms of the underlying exact sampling distribution theory, which greatly helps to clarify the assumptions underlying the Ancova model, to understand the nature and limitations of its conclusions, and to correct various misconceptions related to its applications. More specifically, significance statements, effect size estimates, and statistical power are all ultimately derived from the statistical distribution theory underlying the Ancova model. In addition, the statistical sampling theory also helps to understand the influence on statistical power of the correlation between the dependent variable and the covariate, and the profound effect of various sampling designs (controlled, randomized, observational) on the conclusions that can be derived from an Ancova.

The aim of the present tutorial is therefore to provide an accessible and succinct account of the sampling theory that underlies the standard statistical Ancova model for comparing two independent groups. Specifically, I explain the application of these sampling distribution results to power computations, to the relation to gain score analyses, and to the effect of the study design. The general discussion will also explain how the contentious issues referred to above are clarified within the more general framework of exact sampling distribution theory.

## 2 The standard Ancova model for comparing two groups

Denote as *y*_*ij*_ the sample values of the dependent variable, and as *x*_*ij*_ the corresponding values of the covariate; the index *i* = 1, 2 denotes the group, and *j* = 1, …, *n* the unit within each group. As in the case of a simple *t*−test, a principal aim then is to test for a difference in the population means of the dependent variable. The specific feature of an Ancova is that it takes the covariate values *x*_*ij*_ into account in order to carry out that test.

**Figure 1:**
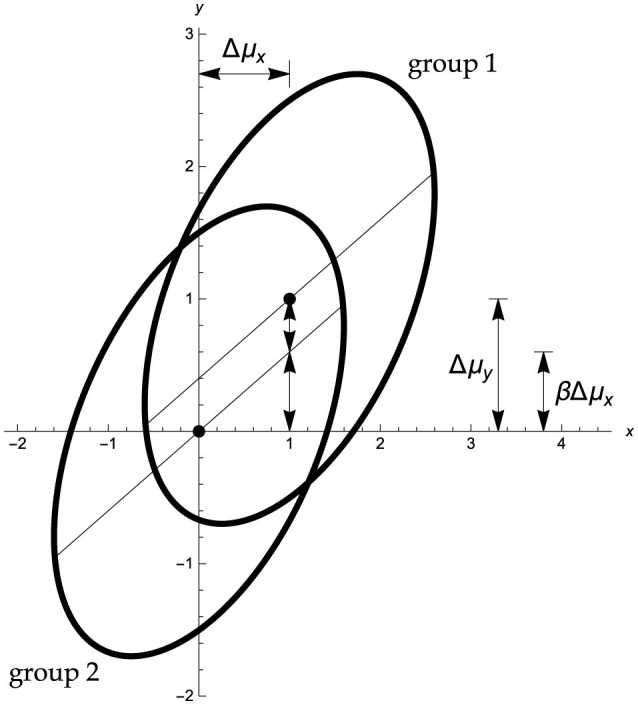
Standard Ancova model for random covariates with standardized parameters Δμ_*y*_ = 1, Δμ_*x*_ = 1, and ϱ = 0.6. Abscissa: covariate *x*, ordinate: dependent variable *y*. Part of the net mean group difference Δμ_*y*_ = 1 in the dependent variable *y* can be explained in terms of the mean group difference in the covariate *x*, namely, βΔ*μ*_*x*_ = 0.6, so that there remains an adjusted mean difference equal to Δμ_*y*_ − βΔ*μ*_*x*_ = 0.4. The H_0_ is that in the population the mean difference in *y* is as predicted, via the regression coefficient β, by the difference in the covariate means, that is, Δμ_*y*_ = βΔ*μ*_*x*_. The Ancova group test evaluates whether the sample $\text{Δ}\bar{y}$ differs from $b\text{Δ}\bar{x}$ by more than chance.

To this end, a number of assumptions are required, as follows. In the standard Ancova model (Figure 1) the dependent variable *y* and the covariate *x* follow, separately in each group, a bivariate normal distribution. The variance–covariance matrix of these distributions is arbitrary but it must be identical in both groups. Let the population variances of the covariate *x* and the dependent variable *y* be denoted as $\sigma_{x}^{2},\sigma_{y}^{2}$, and their within-group correlation as ϱ. These assumptions imply that in both populations the slope of the regression of *y* on *x* is equal to $\beta =\varrho \frac{\sigma_{y}}{\sigma_{x}}$. The population means of *x, y* in group 1 are μ_*x*_1__, μ_*y*_1__, respectively, and they are μ_*x*_2__, μ_*y*_2__ in group 2. It is convenient to denote as Δμ_*y*_ = μ_*y*_1__ − μ_*y*_2__, and correspondingly Δμ_*x*_ = μ_*x*_1__ − μ_*x*_2__. In the following this set of standard assumptions is referred to as the *bivariate normal Ancova model* (cf., Schneider et al., 2015; Winer et al., 1991, p. 770ff).

In an alternative Ancova model variant the covariates *x*_*ij*_ are considered to be fixed (e.g., Cohen, 2009, ch. 9; Maxwell et al., 2018, ch. 9). Significance statements and parameter estimation then refer to hypothetical replications always using the same, given values *x*_*ij*_ of the covariate. This fixed-covariate model may be interpreted as a special case of the model in Figure 1, such that it corresponds to the bivariate normal model but conditional on the realization of a specific set of values *x*_*ij*_. While the fixed and random covariate frameworks test the same hypotheses, the difference between them is crucial for power analysis and sample-size planning (cf., Shieh, 2017, 2021). The fixed-covariate model is plausible, for example, when the covariate is limited to a small set of fixed values which are under the control of the investigator (e.g., when the participants of a study comparing two drugs are paid 100, 125, 150, 175, or 200 USD for their participation), and the intended inference is limited to these specific values. In more typical Ancova applications such as studies A and B, though, the investigator has no direct control over the covariate values, and then the appropriate assumption clearly is that hypothetical replications will generate a different set of covariate values, as assumed under the bivariate normal model. In many Ancova applications, the dependent variable and the covariate actually measure the same variable before and after a treatment; it is then inconsistent to treat the first measurement of that variable as fixed but the second as random.

The basic conceptual logic underlying an Ancova group comparison is simple, even if this simplicity tends to be clouded by technical details. Suppose the dependent variable is related to the covariate in the manner shown qualitatively in Figure 1, and that the treatment exerts no genuine effect of its own. Then we would expect that the group means of *y* differ by an amount that is proportional to the group difference in the covariate, as indicated by the within-group regression slope. To the degree that the actually observed difference in the group means of *y* differs from that prediction, there is evidence that the treatment adds a separate effect that can not simply be ascribed to differences in the covariate.

A central consideration of the Ancova is that even when the treatments are allocated at random to the units (so that μ_*x*_1__ = μ_*x*_2__, as would be expected in Study A) in any particular sample the means of the covariate will practically never be exactly the same in the two groups, just as even a perfectly symmetric coin will in 100 tosses rarely land on head and tail exactly 50 times. There will then nearly always be some imbalance in the sample with respect to the covariate, and if differences in the covariate are associated with differences in the dependent variable, the comparison is biased. This point applies with even more force when there are systematic differences with respect to the covariate between the two groups (i.e., when μ_*x*_1__ ≠ μ_*x*_2__), as would be expected in non-randomized studies using intact (pre-existing) groups, such as Study B above. An Ancova seeks to compare the means of the dependent variable based on a statistical adjustment of these potential imbalances.

In the notation above, the population regressions (i.e., the conditional expectation of the dependent variable *y*, given a specific covariate value of *x*) for the groups are


(1.a) $$\begin{array}{l} {\hat{y}}_{1}=\left(\mu_{y_{1}}-\beta \mu_{x_{1}}\right)+\beta x \end{array}$$

(1.b) $$\begin{array}{l} {\hat{y}}_{2}=\left(\mu_{y_{2}}-\beta \mu_{x_{2}}\right)+\beta x \end{array}$$

The assumption of a common variance-covariance matrix implies that the regression lines in the two groups are parallel, as shown in Figure 1. Thus, for any specific value *x* of the covariate the expected difference in the dependent variable, that is, the vertical separation of the regression lines ${\hat{y}}_{1}$ and ${\hat{y}}_{2}$ in Figure 1, is independent of *x*, and equals


(2) $$\begin{array}{l} {\hat{y}}_{1}-{\hat{y}}_{2}=\left(\mu_{y_{1}}-\beta \mu_{x_{1}}\right)-\left(\mu_{y_{2}}-\beta \mu_{x_{2}}\right)\\ \;=\left(\mu_{y_{1}}-\mu_{y_{2}}\right)-\beta \left(\mu_{x_{1}}-\mu_{x_{2}}\right)=\text{Δ}\mu_{y}-\beta \text{Δ}\mu_{x} \end{array}$$

If the treatment has no effect in the population then we expect no difference in the dependent variable for any given value *x* of the covariate. In view of Equation 2, the H_0_ of the Ancova group comparison thus states that in the population


(3) $$\begin{array}{l} \text{Δ}\mu_{y}=\beta \text{Δ}\mu_{x} \end{array}$$

The Ancova between-group test evaluates if the corresponding sample estimates $\text{Δ}\bar{y}$ and $b\text{Δ}\bar{x}$ differ by more than chance.

Note especially that the H_0_ tested by an Ancova is *not* that the marginal means of the dependent variable are equal, μ_*y*_1__ = μ_*y*_2__ (i.e., Δμ_*y*_ = 0), as would be the case in a standard *t*−test ignoring the covariate *x*. Rather, the H_0_ in an Ancova group comparison is that the means of the dependent variable are equal after they have been adjusted for imbalances of the covariate. Specifically, in Study B the H_0_ tested by an Ancova asserts that male and female graduates of the same height would on average show the same performance.

Two special cases illustrate the nature of the logic of adjustment underlying an Ancova particularly well. In a randomized design we must have Δμ_*x*_ = 0, as each unit is equally likely to receive the one or other treatment. Thus, differences in the covariate between the two groups are unsystematic, and will arise only on a random basis, as in Study A. Even if random, in any specific sample there will still arise a nonzero difference $\text{Δ}\bar{x}$ of the covariate sample means, and the Ancova then adjusts the comparison of the means of the dependent variable for this random component.

Another special case arises when the covariate is unrelated to the dependent variable, ϱ = 0, which implies that the population regression slope β = 0. There may then well be (random or systematic) differences between the two groups regarding the covariate but these differences would not systematically influence the comparison of the means of the dependent variable. Again, even if *x* and *y* are unrelated in the population, in any specific sample the slope estimate *b* would typically not be equal to zero, and an adjustment would be applied to compare the means. As explained below, if ϱ = 0 this adjustment adds a noise component that reduces the sensitivity of the comparison, relative to an analysis igoring the covariate altogether.

## 3 Sample statistics and main distributional results

Let the sample means of the covariate and the dependent variable in group *i* = 1, 2 be denoted as ${\bar{x}}_{i}$ and ${\bar{y}}_{i}$, respectively. Their differences in the sample are


(4.a) $$\begin{array}{l} \text{Δ}\bar{x}={\bar{x}}_{1}-{\bar{x}}_{2} \end{array}$$

(4.b) $$\begin{array}{l} \text{Δ}\bar{y}={\bar{y}}_{1}-{\bar{y}}_{2} \end{array}$$

In the usual summation notation the sample sums of squares and products are


(5.a) $$\begin{array}{l} s_{xx}=\overset{2}{\underset{i=1}{\sum }}\overset{n}{\underset{j=1}{\sum }}{\left(x_{ij}-{\bar{x}}_{i}\right)}^{2} \end{array}$$

(5.b) $$\begin{array}{l} s_{yy}=\overset{2}{\underset{i=1}{\sum }}\overset{n}{\underset{j=1}{\sum }}{\left(y_{ij}-{\bar{y}}_{i}\right)}^{2} \end{array}$$

(5.c) $$\begin{array}{l} s_{xy}=\overset{2}{\underset{i=1}{\sum }}\overset{n}{\underset{j=1}{\sum }}\left(x_{ij}-{\bar{x}}_{i}\right)\left(y_{ij}-{\bar{y}}_{i}\right) \end{array}$$

Note that all sums are taken relative to the means of their respective group, *i*. The sample estimate of the (common) slope β, and the sample estimate of the (common) squared correlation ϱ^2^ are then


(6) $$\begin{array}{lll} b=\frac{s_{xy}}{s_{xx}} & \text{and} & r^{2}=\frac{s_{xy}^{2}}{s_{xx}\cdot s_{yy}} \end{array}$$

The mean squared error of the bivariate normal Ancova model is estimated in the sample as


(7) $$\begin{array}{l} \text{MSE}=\left(1-r^{2}\right)\cdot \frac{s_{yy}}{2n-3} \end{array}$$

which is nearly always, and often considerably, smaller than the MSE of $\frac{s_{yy}}{2n-2}$ in an Anova of the dependent variable.

The sample estimate of the mean squared treatment effect, adjusted for the covariate is


(8) $$\begin{array}{l} \text{MST}=\frac{{\left(\text{Δ}\bar{y}-b\text{Δ}\bar{x}\right)}^{2}}{\frac{2}{n}\left[1+\frac{n}{2}\frac{{\left(\text{Δ}\bar{x}\right)}^{2}}{s_{xx}}\right]}=n\left(n-1\right)\cdot \frac{{\left(\text{Δ}\bar{y}-b\text{Δ}\bar{x}\right)}^{2}}{2\left(n-1\right)+f} \end{array}$$

Note that if the covariate means do not differ in the two samples ($\text{Δ}\bar{x}=0$) then MST reduces to the standard expression $\frac{n}{2}\cdot {\left(\text{Δ}\bar{y}\right)}^{2}$ used in the analysis of variance. The conceptually important quantity *f* in Equation 8 is defined as


(9) $$\begin{array}{l} f=n\left(n-1\right)\cdot \frac{{\left(\text{Δ}\bar{x}\right)}^{2}}{s_{xx}}=\frac{n}{2}\cdot {\left(\frac{\text{Δ}\bar{x}}{s_{x}}\right)}^{2} \end{array}$$

where $s_{x}^{2}=s_{xx}/\left[2\left(n-1\right)\right]$ is the usual unbiased sample estimate of the variance $\sigma_{x}^{2}$; note that $\frac{\text{Δ}\bar{x}}{s_{x}}$ is Cohen's standardized mean difference *d*_*p*_ (Goulet-Pelletier and Cousineau, 2018). According to Equation 9, the sample statistic *f* is equal to the value of the standard statistic $t_{2\left(n-1\right)}^{2}=F_{1,2\left(n-1\right)}$ used to compare the covariate means ${\bar{x}}_{1}$ and ${\bar{x}}_{2}$ in the two groups^1^. It thus measures the imbalance of the sample covariate means between the two groups; according to Equation 8, large values of *f* will generally reduce the value of MST. As a rough rule of thumb, for *n* > 5 a value of about *f* > 5 suggests that the covariate means differ by more than chance.

The *F*−statistic for the comparison of the adjusted group means is, as usual, computed in the sample as the ratio of the treatment and error mean squares


(10) $$\begin{array}{l} F_{1,2n-3}=\frac{\text{MST}}{\text{MSE}} \end{array}$$

If the H_0_ described in Equation 3 is true, that is, if Δμ_*y*_ = βΔ*μ*_*x*_, then the sample statistic defined in Equation 10 has a central *F*_1, 2*n*−3_−distribution. This result ensures that we can compare the sample statistic *F*_1, 2*n*−3_ to the critical *F*−value at the desired level of significance. As explained below, this remains true even when the covariates *x*_*ij*_ are considered as fixed values.

Alternatively, the *t*−statistic for the adjusted mean difference takes the standard form


(11) $$\begin{array}{l} t_{2n-3}=\frac{\text{Δ}\bar{y}-b\text{Δ}\bar{x}}{\text{s.e.}\left(\text{Δ}\bar{y}-b\text{Δ}\bar{x}\right)} \end{array}$$

and the standard error of the adjusted mean difference is estimated in the sample as


(12) $$\begin{array}{l} \text{s.e.}\left(\text{Δ}\bar{y}-b\text{Δ}\bar{x}\right)=\sqrt{\text{MSE}\cdot \frac{2}{n}\cdot \left[1+\frac{n}{2}\cdot \frac{{\left(\text{Δ}\bar{x}\right)}^{2}}{s_{xx}}\right]} \end{array}$$

(for related results, see Großand Möller, 2024). Again, if Δμ_*y*_ = βΔ*μ*_*x*_, then the sample statistic defined in Equation 11 has a central *t*_2*n*−3_−distribution. From Equations 7, 8, the statistic *F*_1, 2*n*−3_ in Equation 10 for the Ancova group comparison is equal to the square of the statistic *t*_2*n*−3_ in Equation 11, and in this sense the two approaches based on *t* or *F* are equivalent.

From Equation 11, the point estimate of the adjusted mean difference is $\text{Δ}\bar{y}-b\text{Δ}\bar{x}$, and its two-sided 1−α confidence interval is computed as


(13) $$\begin{array}{l} {\text{C.I.}}_{1-\alpha }=\left(\text{Δ}\bar{y}-b\text{Δ}\bar{x}\right)\pm t_{2n-3}\left(\alpha /2\right)\cdot \text{s.e.}\left(\text{Δ}\bar{y}-b\text{Δ}\bar{x}\right) \end{array}$$

The H_0_:Δμ_*y*_ = βΔ*μ*_*x*_ is rejected by the *t*− or the *F*−test at the significance level α if and only if the confidence interval C.I._1−α_ does not contain the value of zero. From Equation 12, the standard error of the adjusted mean difference increases with the sample imbalance $\text{Δ}\bar{x}$ of the covariate between the two groups, and when $\text{Δ}\bar{x}=0$ it reduces to the standard form for the difference of two means.

We next turn to the expectation and distribution of these sample statistics under the bivariate normal model in the general case, that is, without assuming the H_0_ to hold; as always, distributional results for the non-null case are needed in order to compute statistical power.

The sample estimate MSE of the mean squared error in Equation 7 is unbiased; its expectation thus equals that part, $\sigma_{e}^{2}$ say, of the variance in the dependent variable not accounted for by the covariate


(14) $$\begin{array}{l} \text{E}\left[\text{MSE}\right]=\left(1-\varrho^{2}\right)\sigma_{y}^{2}=\sigma_{e}^{2} \end{array}$$

The sample estimate MST of the adjusted treatment mean square in Equation 8 depends on the individual covariate values *x*_*ij*_ only through the mean difference $\text{Δ}\bar{x}$ and the sample statistic *f* as defined in Equation 9. Given *f*, the conditional expectation of MST is (cf., Sprott, 1970, Equation 5; Schneider et al., 2015, Equation B11)


(15) $$\begin{array}{l} \text{E}\left[\text{MST}|f\right]={\left(\text{Δ}\mu_{y}-\beta \text{Δ}\mu_{x}\right)}^{2}\cdot \frac{n\left(n-1\right)}{2\left(n-1\right)+f}+\sigma_{e}^{2} \end{array}$$

As expected, if the H_0_:Δμ_*y*_ = βΔ*μ*_*x*_ tested by the Ancova holds, then E[MST|*f*] reduces to $\sigma_{e}^{2}$, the expected MSE. Note that if *f* = 0, then E[MST|*f*] will exceed $\sigma_{e}^{2}$ by the amount $\frac{n}{2}{\left(\text{Δ}\mu_{y}-\beta \text{Δ}\mu_{x}\right)}^{2}$. Recall that the statistic *f* evaluates the sample imbalance of the covariate between the two groups. To the degree that *f* is greater than zero it will reduce the amount by which E[MST|*f*] exceeds $\sigma_{e}^{2}$, that is, it will reduce power.

In the bivariate normal Ancova model the covariate values *x*_*ij*_ have a normal marginal distribution within each group, with possibly different means but equal variance. Therefore, the sample statistic *f* defined in Equation 9 is distributed as a noncentral *F*−variate


(16) $$\begin{array}{l} f\sim F_{1,2\left(n-1\right),\text{λ}} \end{array}$$

where the noncentrality parameter λ has the standard form


(17) $$\begin{array}{l} \text{λ}=\frac{n}{2}\cdot {\left(\frac{\text{Δ}\mu_{x}}{\sigma_{x}}\right)}^{2} \end{array}$$

If the expected values of the covariate do not differ between the groups—for example, because a randomized design, as in Study A above, is used—then Δμ_*x*_ = 0, and in this case *f* has a *central* *F*−distribution, that is, λ = 0.

Integrating Equation 15 across the density of *f*, the unconditional expected mean square of the treatment is (cf., Schneider et al., 2015, Equation B13)


(18) $$\begin{array}{l} \text{E}\left[\text{MST}\right]={\left(\text{Δ}\mu_{y}-\beta \text{Δ}\mu_{x}\right)}^{2}\cdot \int_{0}^{\infty }\frac{n\left(n-1\right)}{2\left(n-1\right)+f}\cdot q_{1,2\left(n-1\right),\text{λ}}\left(f\right)df+\sigma_{e}^{2} \end{array}$$

where *q*_1, 2(*n*−1), λ_ is the density of *f*, that is, of a noncentral *F*_1, 2(*n*−1), λ_, with noncentrality parameter λ. Note that *f* depends only on the covariates *x*_*ij*_, and so the integral in Equation 18 depends only on the marginal distribution of the covariates *x*_*ij*_, and is independent of the parameters $\mu_{y_{i}},\sigma_{y}^{2}$, and ϱ of the basic model in Figure 1. In essence, it is a multiplier of ${\left(\text{Δ}\mu_{y}-\beta \text{Δ}\mu_{x}\right)}^{2}$, defined by the features, as summarized by λ, of the (normal) marginal distribution of the covariate. It may be shown that if Δμ_*x*_ = 0, as in randomized designs, then E[MST] reduces to $\sigma_{e}^{2}+\frac{n\left(n-1\right)}{2n-1}\cdot {\left(\text{Δ}\mu_{y}\right)}^{2}$.

The conditional distribution function of the Ancova sample *F*−value, given the statistic *f*, may be written as


(19) $$\begin{array}{l} P\left(F=\frac{\text{MST}}{\text{MSE}}\leq t|f\right)=P\left(F_{1,2n-3,k}\leq t|f\right) \end{array}$$

where *F*_1, 2*n*−3, *k*_ follows a noncentral *F*−distribution with *df* equal to 1 and 2*n*−3, and noncentrality parameter *k*, defined by


(20) $$\begin{array}{l} k=k\left(f\right)=n\left(n-1\right)\frac{{\left(\frac{\text{Δ}\mu_{y}-\beta \text{Δ}\mu_{x}}{\sigma_{e}}\right)}^{2}}{2\left(n-1\right)+f} \end{array}$$

If the sample means of the covariate are equal ($\text{Δ}\bar{x}=0$) then *f* = 0, and the noncentrality parameter *k* reduces to the standard form $\frac{n}{2}\cdot {\left(\frac{\text{Δ}\mu_{y}-\beta \text{Δ}\mu_{x}}{\sigma_{e}}\right)}^{2}$, whereas greater values of *f* will reduce the value of *k*.

The result Equation 19 is conditional on the *x*_*ij*_, as summarized by *f*. As the noncentrality parameter *k* = *k*(*f*) in Equation 19 depends on the statistic *f*, we get the unconditional distribution of the sample *F* statistic by integrating the conditional distribution function across the density *q*_1, 2(*n*−1), λ_ of *f*, which leads to a mixture of the family *F*_1, 2*n*−3, *k*_ across its noncentrality parameter *k*. The unconditional distribution function of the sample *F* statistic may thus be represented as


(21) $$\begin{array}{l} P\left(F=\frac{\text{MST}}{\text{MSE}}\leq t\right)=\int_{0}^{\infty }P\left(F_{1,2n-3,k\left(f\right)}\leq t|f\right)\cdot q_{1,2\left(n-1\right),\text{λ}}\left(f\right)df \end{array}$$

where *q*_1, 2(*n*−1), λ_ is as above. In essence, the sample *F*−value follows a mixture of noncentral *F*−distributions, with a mixing distribution that is itself a noncentral *F*−distribution, namely, *q*_1, 2(*n*−1), λ_.

Equation 21 is a central result that may be used to compute statistical power in the general case under the bivariate normal model (cf., Shieh, 2017, 2021). An important general implication of Equation 21 is that in an Ancova with random covariates the sample *F* statistic does *not* follow a standard noncentral *F*−distribution but has the more complex mixture structure indicated in Equation 21. If, in contrast, the covariates *x*_*ij*_ are considered as fixed (e.g., Cohen, 2009, ch. 8.3.5), then one would use the conditional distribution *P*(*F*_1, 2*n*−3, *k*_ ≤ *t*|*f*) of Equation 19, where the noncentrality parameter *k* = *k*(*f*) defined by Equation 20 is determined by the value of *f* as given by the fixed *x*_*ij*_ in the way prescribed by Equation 9.

Note, however, that the noncentrality parameter *k* = *k*(*f*) given in Equation 20 is generally zero if the null hypothesis Δμ_*y*_ = βΔ*μ*_*x*_ holds, independent of the sample statistic *f*, which depends only on the covariates *x*_*ij*_. In this case, in Equation 21 the first factor of the integrand (which then becomes a central *F*−distribution function, *k* = 0, with *df* of 1 and 2*n*−3) may be taken before the integral sign, and the remaining integral across the density *q*_1, 2(*n*−1), λ_ necessarily evaluates to 1. Accordingly, in the null case of H_0_:Δμ_*y*_ = βΔ*μ*_*x*_ the empirical Ancova *F* will always follow the *central* *F*_1, 2*n*−3_−distribution, both with fixed and with random covariates. However, in the non-null case Δμ_*y*_ ≠ βΔ*μ*_*x*_ the computation of statistical power or required sample sizes must be based on Equation 21 when the covariates vary randomly, or on Equation 19 when the covariates are considered as fixed (cf., Shieh, 2017, 2021).

## 4 A numerical example

**Figure 2:**
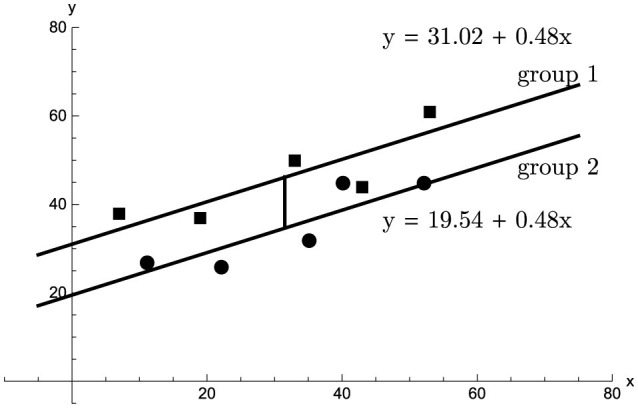
Two groups (group 1: squares, group 2: dots) comprising *n* = 5 participants each were measured on the dependent variable *y* (ordinate) and on the covariate *x* (abscissa). The adjusted mean difference, equal to 11.48, is shown as the vertical separation of the parallel regression lines.

These results can be illustrated using the fictitious data set shown in Table 1, Figure 2.

**Table 1:** Two groups comprising *n* = 5 participants each were measured on the dependent variable, *y*, and on the covariate, *x*.

**Participant**	**Group 1**		**Group 2**	
**within group**	**covariate** *x*	**dep. variable** *y*	**covariate** *x*	**dep. variable** *y*
1	43	44	52	45
2	33	50	11	27
3	19	37	22	26
4	7	38	40	45
5	53	61	35	32
means	31	46	32	35

The data set is shown in Figure 2.

Two groups comprising *n* = 5 participants each were measured on the dependent variable *y* and on the covariate *x*. The sample covariate means are ${\bar{x}}_{1}=31$ and ${\bar{x}}_{2}=32$, a difference of $\text{Δ}\bar{x}=-1$. The sample means of the dependent variable are ${\bar{y}}_{1}=46$ and ${\bar{y}}_{2}=35$, so that $\text{Δ}\bar{y}=11$. The sample sums of squares and products are *s*_*xx*_ = 2366, *s*_*yy*_ = 744, and *s*_*xy*_ = 1143. From these values by Equation 6 we have *b* = 0.483 and *r*^2^ = 0.742. Thus, there is a positive relation between *x* and *y*, and a slight covariate imbalance favoring group 2. The estimate of the adjusted mean difference, shown as a vertical line in Figure 2, equals $\text{Δ}\bar{y}-b\cdot \text{Δ}\bar{x}=11.48$, and by Equations 12, 13 the corresponding 95%−confidence interval is [3.66, 19.30]. Using Equations 7, 8, 10 the Ancova group comparison sample value *F*_1, 7_ = 12.02, giving *p* =.011. By comparison, for the same data set an Anova of the change scores produces *F*_1, 8_ = 3.49, *p* =.099, and an Anova of the dependent variable alone, disregarding the covariate altogether, gives *F*_1, 8_ = 3.25, *p* =.109.

## 5 General discussion

What exactly can be concluded under the bivariate normal Ancova model shown in Figure 1 from a significant *F*−value computed from the sample data in the way indicated by Equations 4–10? Regardless of the design used, a significant sample *F* indicates, at the chosen level of significance, that the mean group difference in the dependent variable, adjusted for (i.e., conditional on) the covariate, is greater than would be expected by chance. A corresponding confidence interval for the adjusted mean difference can be computed as indicated in Equation 13, which will, in a proportion of 1−α of all samples, cover the true adjusted mean difference for any set of parameters of the model in Figure 1, including, specifically, the case in which Δμ_*x*_ ≠ 0 (cf., Senn, 2006). This most basic form of an Ancova-related conclusion addresses the purely *statistical* question of whether the observed adjusted mean difference is greater than the error margin, and not the question of how to *interpret* any such difference. It essentially states *that* the adjusted means differ systematically, but—up to ruling out the effect of the covariate—it leaves open *why* they differ.

As in other research contexts, more specific causal interpretations of significant results depend critically on the design of the study (e.g., Huitema, 2011, ch. 8). Specifically, under a nonrandomized design, even a significant mean difference, adjusted for the chosen covariate, can still be related to other, uncontrolled covariates rather than to the factor defining the two groups. In the case of Study B, even if male and female graduates of the same height differ in the maximum height jumped over, this adjusted effect might still be unrelated to gender per se, but may reflect, for example, that better training opportunities were provided for male graduates. In a nonrandomized design, an Ancova clearly provides no firm basis to attribute a significant adjusted mean difference in high jump performance, specifically, to the group variable gender, even though it does provide a valid evaluation of the statistical significance of the adjusted mean group difference (for a recent account of how to interpret effects in observational designs in terms of acyclic directed graphs, see Cinelli et al., 2024). On the other hand, in the context of a randomized study a significant difference in the adjusted means can be attributed specifically to the factor that defines group membership. For example, in the randomized Study A, a significant mean difference in drinking time, adjusted for the urge to consume alcoholic beverages, leads to the more specific interpretation that the glass shape was the cause of the adjusted differences observed.

These main forms of potential conclusions that can be drawn from an Ancova are complemented by various aspects related to the application and interpretation of an Ancova, which I discuss next in turn.

## 6 The standardized Ancova model

It is readily seen from the basic distributional features, such as Equations 9, 17, 20, that as far as significance and power are concerned the bivariate normal Ancova model may be standardized with no change of probability statements. Specifically, we may set μ_*x*_2__ = μ_*y*_2__ = 0 and σ_*x*_ = σ_*y*_ = 1 in which case only three effective parameters of the standardized population model remain. These three parameters are the standardized distance Δμ_*y*_ of the marginal group means in the dependent variable *y*, the standardized distance Δμ_*x*_ of the marginal group means in the covariate *x*, and the correlation ϱ of *x* and *y* which in the standardized model is equal to the within-group regression slope β. Therefore, any parameter combination within the general bivariate normal population model may be reduced to the more manageable standardized Ancova model without affecting power and significance.

## 7 Statistical power as a function of the correlation between the covariate and the dependent variable

Equation 21 may be used to address the practically relevant question: Which correlation ϱ should the covariate and the dependent variable ideally have in order to maximize power? To address this point succinctly, I will use the standardized formulation of the bivariate normal Ancova model. As we will see, the answer depends on whether the study is randomized (so that Δμ_*x*_ = 0, as in Study A) or not (Δμ_*x*_ ≠ 0, as in Study B).

In principle, increasing the correlation ϱ has two quite separate effects. First, by Equations 7, 14 an increase in ϱ reduces the error variance $\sigma_{e}^{2}=\sigma_{y}^{2}\left(1-\varrho^{2}\right)$, which by Equation 10 in turn increases the empirical *F*_1, 2*n*−3_−values for the adjusted group comparison, and thus power. Second, all other aspects equal, an increase of ϱ also increases the regression slope $\beta =\varrho \frac{\sigma_{y}}{\sigma_{x}}$, and thus typically reduces the size Δμ_*y*_−β·Δμ_*x*_ of the adjusted mean difference (cf., Figure 1). Put simple, more of the total increase Δμ_*y*_ in mean *y* may then be explained on the basis of the increase Δμ_*x*_ in mean *x*. The first of these counteracting effects will tend to be dominant if Δμ_*x*_ is small or zero, but if Δμ_*x*_ is medium or large then increasing ϱ may entail a considerable loss of power. I discuss these two scenarios separately.

**Figure 3:**
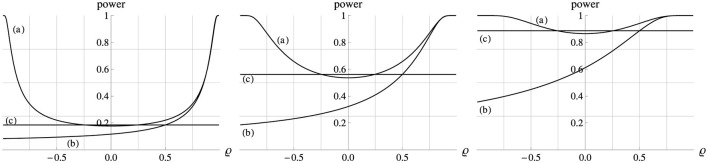
Power (ordinate) of the Ancova group comparison (a) and of a gain score analysis (b) as a function of ϱ (abscissa). In all plots Δμ_*x*_ = 0, σ_*x*_ = σ_*y*_ = 1, *n* = 10, and α = 0.05. Left, middle, right panel: Δμ_*y*_ = 0.5, 1.0, 1.5. The horizontal lines (c) show the power of the corresponding *t*−test of the means of the dependent variable, ignoring the covariate altogether. For Δμ_*x*_ = 0 all three analyses test the same H_0_:Δμ_*y*_ = 0.

If Δμ_*x*_ = 0 then for any ϱ (and thus any β) Equation 3 for the Ancova group comparison reduces to testing H_0_:Δμ_*y*_ = 0. Figure 3 indicates that for Δμ_*x*_ = 0 statistical power then generally increases as ϱ^2^ increases. For ϱ = 0 power is slightly lower than that of an independent *t*−test that ignores the covariate altogether. The reason is that for ϱ = 0 the regression-based Ancova adjustment of the sample means leads to a loss of one degree of freedom, and adds a noise component that reduces the sensitivity of the comparison. This effect is typically small unless *n* is very small, and it is soon compensated and then superseded as ϱ^2^ increases. Thus, in a randomized study a covariate that correlates strongly with the dependent variable within each group will be useful in increasing power.

**Figure 4:**
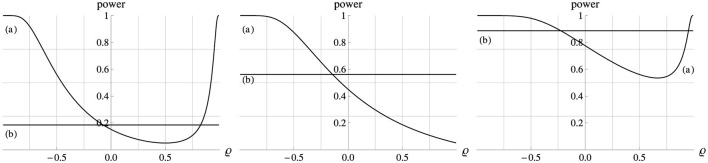
Power (ordinate) of the Ancova group comparison (a) as a function of ϱ (abscissa). In all plots Δμ_*x*_ = 1, σ_*x*_ = σ_*y*_ = 1, *n* = 10, and α = 0.05. Left, middle, right panel: Δμ_*y*_ = 0.5, 1.0, 1.5. The horizontal lines (b) show the power of the corresponding *t*−test of the means of the dependent variable, ignoring the covariate altogether.

These simple relations become considerably more complex if Δμ_*x*_ ≠ 0, as illustrated in Figure 4 for the case of Δμ_*x*_ = 1. By Equation 3, the Ancova group comparison tests if Δμ_*y*_−βΔ*μ*_*x*_ = 0, and in the standardized model version we have β = ϱ. Therefore, the H_0_ holds if $\varrho =\frac{\text{Δ}\mu_{y}}{\text{Δ}\mu_{x}}$, at which point the power function reaches its minimum, the level α.

For example, in the left panel of Figure 4 (Δμ_*y*_ = 0.5) the adjusted mean difference Δμ_*y*_ − βΔ*μ*_*x*_ falls to the H_0_ value of zero at ϱ = β = 0.5, at which point the power curve reaches the minimum value of α =.05. In the middle panel (Δμ_*y*_ = 1.0) that minimum is just reached at the maximum correlation, ϱ = 1.0, when Δμ_*y*_ − βΔ*μ*_*x*_ = 0, so that in this scenario power generally decreases as the correlation ϱ increases. In the right panel of Figure 4 (Δμ_*y*_ = 1.5) the adjusted mean difference Δμ_*y*_ − βΔ*μ*_*x*_ remains positive even for the strongest correlation but it declines as ϱ = β increases. However, beyond ϱ = 0.67 this decline is more than compensated by the simultaneous reduction of the error variance $\sigma_{e}^{2}$, so that power increases again beyond that point. In summary, to maximize power in a nonrandomized study design (Δμ_*x*_ ≠ 0) it is critical to select a covariate whose correlation with the dependent variable is not close to $\varrho =\frac{\text{Δ}\mu_{y}}{\text{Δ}\mu_{x}}$ in the neighborhood of which the power curve drops to a minimum of α.

## 8 Ancova and gain score analysis

In some study designs to which an Ancova is applied, the covariate and the dependent variable are commensurate—typically measuring the same variable before and after a treatment. In such cases, a popular alternative to an Ancova is the comparison between groups of the mean difference, or *gain*, scores ${\bar{y}}_{i}-{\bar{x}}_{i}$, and the hypothesis being tested is that in the population the mean gain scores are the same in both groups. In the present notation, an equivalent formulation of this hypothesis is H_0_:Δμ_*y*_ = Δμ_*x*_. A gain score analysis tests this H_0_ by a *t*−test or an Anova comparing the mean gain scores between the two groups. In the latter case, the sample *F*−value has 1 and 2(*n*−1) degrees of freedom, and the associated noncentrality parameter is readily shown to be


(22) $$\begin{array}{l} \theta =\frac{n}{2}\cdot {\left(\frac{\text{Δ}\mu_{y}-\text{Δ}\mu_{x}}{\sigma_{y-x}}\right)}^{2} \end{array}$$

where $\sigma_{y-x}^{2}=\sigma_{x}^{2}+\sigma_{y}^{2}-2\varrho \sigma_{x}\sigma_{y}$. Note that as ϱ decreases the noncentrality parameter θ – and thus the statistical power of a gain score analysis – generally decreases.

It is essential to appreciate the relationship between an Ancova and a gain score analysis (e.g., van Breukelen, 2006; Samuels, 1986; Senn, 2006). The formulation of the H_0_:Δμ_*y*_ = Δμ_*x*_ underlines the central point, first emphasized by Lord (1967), that in general a gain score analysis and an Ancova test *different* hypotheses, each of which may or may not be rejected for any given data set. Specifically, an Ancova focuses on (the differences in) *conditional* means—namely, of *y*, given *x*—whereas a gain score analysis focuses on (the differences in) *marginal* means.

Comparing the H_0_:Δμ_*y*_ = Δμ_*x*_ of a gain score analysis to the corresponding Ancova formulation H_0_:Δμ_*y*_ = βΔ*μ*_*x*_ in Equation 3, we see that under the general bivariate normal model in Figure 1 the two hypotheses tested will coincide if either Δμ_*x*_ = 0 (i.e., under a randomized design) or β = 1. Note that although the *hypothesis* tested under those two scenarios is the same, the *test statistic* computed from the sample in a gain score analysis and in an Ancova is not, as can be seen from the fact that the sample *F*−values have (1, 2*n*−2) degrees of freedom for a gain score analysis, and (1, 2*n*−3) for an Ancova. Also note that under the model in Figure 1, the gain scores in both groups are in any case—not just for β = 1 or Δμ_*x*_ = 0 – normally distributed with equal variance, and thus satisfy the formal requirements of a valid *t*− or *F*−test. Figure 3 illustrates that when the variances of the covariate and the dependent variable are equal ($\sigma_{x}^{2}=\sigma_{y}^{2}$) then for all correlation levels and for all effect sizes, an Ancova is more powerful than a gain score analysis under a randomized design (i.e., Δμ_*x*_ = 0). When the variances are unequal ($\sigma_{x}^{2}\neq \sigma_{y}^{2}$), which is necessarily the case when $\beta =\varrho \frac{\sigma_{y}}{\sigma_{x}}=1$, then an Ancova is not uniformly more powerful than a gain score analysis.

## 9 Power as a function of the study design

Equations 20, 21 also explain why the statistical power of an Ancova depends critically on the study design. Specifically, the study design determines the noncentrality parameters *k* in Equation 20 and λ in Equation 17, and according to Equation 21 power will increase with *k* and decrease with λ.

In a *controlled* design, the researcher choses deliberately all values *x*_*ij*_ of the covariate, typically such that ${\bar{x}}_{1}={\bar{x}}_{2}$. Therefore, in Equation 9 the quantity *f* = 0 *in each sample*. As indicated by Equation 19 for *f* = 0, the Ancova sample statistic *F*_1, 2*n*−3_ as defined in Equation 10 then follows a standard noncentral *F*−distribution, with a noncentrality parameter *k* that takes, by Equation 20, the simplified standard form $k=\frac{n}{2}\cdot {\left(\frac{\text{Δ}\mu_{y}}{\sigma_{e}}\right)}^{2}$.

In a *randomized* design, the units are assigned at random to their treatment group (cf., Study A). In this case Δμ_*x*_ = 0; the sample means ${\bar{x}}_{1}$ and ${\bar{x}}_{2}$ will then be equal, not in each individual sample, but *in expectation*, which implies λ = 0. In a randomized design the Ancova sample statistic *F*_1, 2*n*−3_ follows no longer a standard noncentral *F*−distribution (as it does under a controlled design), but rather a mixture of noncentral *F*−distributions, and the noncentrality parameter *k* in Equation 20 then takes the form $k=\frac{n\left(n-1\right)}{2\left(n-1\right)+f}\cdot {\left(\frac{\text{Δ}\mu_{y}}{\sigma_{e}}\right)}^{2}$ which is smaller than in a controlled design, indicating some loss of power.

In an *observational* design, the researcher has no control over the covariates, and compares pre-existing (“intact”) groups, in which typically Δμ_*x*_ ≠ 0, which implies λ>0. In this case, the full form given in Equation 20 applies, and the noncentrality parameter *k* will in typical applications be smaller than in controlled or randomized designs. This is because the sample statistic *f* measuring the covariate imbalance tends to be larger in an observational design, which by Equation 20 reduces *k*. Thus, other things equal the power of the Ancova group comparison is largest in controlled designs with ${\bar{x}}_{1}={\bar{x}}_{2}$ (and so *f* = 0), it is intermediate for randomized designs (μ_*x*_1__ = μ_*x*_2__), and it is lowest with observational designs (μ_*x*_1__ ≠ μ_*x*_2__).

## 10 Conditional vs. marginal means

As indicated by Equations 1, 2, the Ancova model compares *conditional* group means, namely, the conditional means of the dependent variable *y* given some specific value *x* of the covariate. In contrast, standard Anova or *t*−tests and gain score analyses compare *marginal* means. This important conceptual distinction is sometimes misunderstood. For example, in their otherwise eminent treatment Schneider et al. (2015, p. 2) state that “it is not so widely-known that the validity of an Ancova also depends on [the] assumption ... that the expected value of the covariate is the same for all of the participants in the experiment”. Accordingly, they conclude (p. 3) that “the statistical [Ancova] test for the between-subject main effect is not valid unless μ_*d*_ = 0” [i.e., Δμ_*x*_ = 0, in the present notation]. The distributional results summarized by Equations 19–Equation 21 clearly indicate that this conclusion is incorrect. Even if Δμ_*x*_ ≠ 0, the statistical Ancova model as shown in Figure 1 correctly tests the H_0_ of equal conditional means at the specified level of significance, and Equation 13 provides a valid confidence interval for the difference in the conditional means.

The argument of Schneider et al. (for a similar view, see, for example, Miller and Chapman, 2001) is based on the erroneous notion that an Ancova compares the *marginal* means of the dependent variable, and not the *conditional* means, given a specific value of the covariate. It is, of course, true that an Ancova does not usually provide a valid statistical test for the equality of the marginal means of the dependent variable, that is, of the H_0_:Δμ_*y*_ = 0. However, as indicated by Equation 3, this hypothesis is in fact only tested by an Ancova if Δμ_*x*_ = 0 (i.e., under a randomized design), or if β = 0, that is, if the dependent variable and the covariate are uncorrelated. In the general case, the equality of the *marginal* means of *y* is simply not the statistical question that an Ancova addresses, and it is misleading to fault Ancova techniques for providing its consistent and valid answers (given the model assumptions are met) aimed at comparing *conditional* means. For example, in Study B an Ancova tests if the mean difference in high jump performance between male and female graduates is larger than expected on the basis of the height differences alone. Even if an Ancova indicates, for example, that male graduates jump on average higher by just the amount predicted from the between-group difference in the covariate height, that would not represent a claim that the marginal mean performance in both groups is equal.

The confusion between marginal and conditional means in Ancova techniques is widespread, even in renowned accounts. For example, Vickers and Altman (2001, p. 1123) state that, when the variables are commensurate, the difference in the sample regression intercepts in the two groups (i.e., the adjusted mean difference) “has a useful interpretation: it is the difference between the mean change scores of each group”, which is incorrect. In the present notation, “the difference between the mean change scores of each group” in the sample is equal to $\text{Δ}\bar{y}-\text{Δ}\bar{x}$. In contrast, the difference of the sample regression intercepts is given by $\text{Δ}\bar{y}-b\cdot \text{Δ}\bar{x}$, which obviously differs from the marginal differences referred to in the statement of Vickers and Altman—even in their own Table 1, where these quantities are equal to 12.7 and 10.8, respectively.

A main limitation of the present tutorial is its restriction to two groups and a single covariate. This deliberate choice is motivated by two considerations: i.) to present the basic distributional features as clearly as possible, and ii.) by the fact that in many areas, Ancova applications typically refer to this most prominent case. The general case involving more groups, unequal group sizes, or more covariates, follows similar distributional principles as outlined above but requires a more elaborate technical apparatus that tends to cloud the intrinsic simplicity of basic Ancova principles. Excellent recent contributions, such as Huitema (2011, ch. 6), Kutner et al. (2004, ch. 22), Maxwell et al. (2018, ch. 9), Schneider et al. (2015), or Shieh (2017, 2021) offer a broader coverage of Ancova techniques.

## Data Availability

The original contributions presented in the study are included in the article/supplementary material, further inquiries can be directed to the corresponding author.

## Footnotes

^1^The notation *t*_*m*_ denotes a *t*−distributed variate with *m* degrees of freedom. Similarly, *F*_*m, n*_ denotes an *F*−distributed variate with *m* numerator and *n* denominator degrees of freedom.

## References

[B1] Attwood A. S. Scott-Samuel N. E. Stothart G. Munafò M. R. (2012). Glass shape influences consumption rate for alcoholic beverages. PLoS ONE. 7:e43007. 10.1371/journal.pone.0043007 22912776 PMC3422221

[B2] Cinelli C. Forney A. Pearl J. (2024). A crash course in good and bad models. Sociol. Methods Res. 53, 1071–1104. 10.1177/0049124122109955233620822

[B3] Cohen J. (2009). Statistical Power Analysis for the Behavioral Sciences (2nd ed.). New York: Psychology Press.

[B4] Goulet-Pelletier J.-C. Cousineau D. (2018). A review of effect sizes and their confidence intervals, part I: the Cohen's *d* family. Quant. Methods Psychol. 14, 242–265. 10.20982/tqmp.14.4.p242

[B5] Groß J. Möller A. (2024). Some additional remarks on statistical properties of Cohen's *d* in the presence of covariates. Statist. Papers 65, 3971–3979. 10.1007/s00362-023-01527-9

[B6] Huitema B. E. (2011). The Analysis of Covariance and Alternatives (2nd ed.). New York: Wiley.

[B7] Kutner M. H. Nachtsheim C. J. Neter J. Li W. (2004). Applied Linear Statistical Models (5th ed.). Chicago: McGraw-Hill.

[B8] Lord F. M. (1967). A paradox in the interpretation of group comparisons. Psychol. Bullet. 68, 304–305. 10.1037/h0025105 6062585

[B9] Maxwell S. E. Delaney H. D. Kelley K. (2018). Designing Experiments and Analyzing Data: A Model Comparison Perspective (3rd ed.). New York, London: Routledge.

[B10] Miller G. A. Chapman J. P. (2001). Misunderstanding analysis of covariance. J. Abnormal Psychol. 110, 40–48. 10.1037/0021-843X.110.1.40 11261398

[B11] Samuels M. L. (1986). Use of analysis of covariance in clinical trials: a clarification. Control. Clini. Trials 7, 325–329. 10.1016/0197-2456(86)90039-5 3802852

[B12] Schneider B. A. Avivi-Reich M. Mozuraitis M. (2015). A cautionary note on the use of the analysis of covariance (Ancova) in classification designs with and without within-subject factors. Front. Psychol. 8:474. 10.3389/fpsyg.2015.00474 25954230 PMC4404726

[B13] Schwarz W. (2008). 40 Puzzles and Problems in Probability and Mathematical Statistics. New York: Springer-Verlag.

[B14] Schwarz W. Reike D. (2018). Regression away from the mean: theory and examples. Br. J. Mathem. Statist. Psychol. 71, 186–203. 10.1111/bmsp.12106 28664975

[B15] Senn S. (2006). Change from baseline and analysis of covariance revisited. Statist. Med. 25, 4334–4344. 10.1002/sim.2682 16921578

[B16] Shieh G. (2017). Power and sample size calculations for contrast analysis in Ancova. Multivariate Behav. Res. 52, 1–11. 10.1080/00273171.2016.1219841 28121163

[B17] Shieh G. (2021). Appraising minimum effect of standardized contrasts in Ancova: statistical power, sample size, and covariate imbalance considerations. Statist. Biopharmaceut. Res. 13, 468–475. 10.1080/19466315.2020.1788982

[B18] Sprott D. A. (1970). Note on Evans and Anastasio on the analysis of covariance. Psychol. Bullet. 73, 303–306. 10.1037/h0028923

[B19] van Breukelen G. J. P. (2006). Ancova vs. change from baseline: more power in randomized studies, more bias in nonrandomized studies. J. Clin. Epidemiol. (2006) 59:1334. 10.1016/j.jclinepi.2006.10.002 16895814

[B20] Vickers A. J. Altman D. G. (2001). Analysing controlled trials with baseline and follow up measurements. Br. Med. J. 323, 1123–1124. 10.1136/bmj.323.7321.1123 11701584 PMC1121605

[B21] Winer B. J. Brown D. R. Michels K. M. (1991). Statistical Principles in Experimental Design (3rd ed.). New York: McGraw-Hill.

